# When is a desmoid not a desmoid? Endometrial cancer as an extracolonic manifestation of MYH Associated Polyposis (MAP)

**DOI:** 10.1186/1897-4287-9-S1-P14

**Published:** 2011-03-10

**Authors:** Cathy Gilpin, Matthew Lines, Eva Tomiak

**Affiliations:** 1 Department of Genetics, Children’s Hospital of Eastern Ontario, Ottawa, Ontario, Canada

## Background

Desmoids are a recognized feature of the classical and attenuated forms of familial adenomatous polyposis (FAP), occurring in 15% of individuals with classic FAP.

## Case history

A 68 year old Caucasian woman presented with a lump in her right groin and lower abdominal pain. No changes in bowel habit or other bowel symptoms were reported. Previous medical history included endometrial cancer diagnosed at age 57, treated by hysterectomy and bilateral salpingo-oophorectomy. The family history was negative for desmoids, colon polyps and cancer.

By CT scan, a 4 x 3 cm soft tissue mass arising from the right rectus muscle and tethered to bowel loops was identified. It was felt to represent a desmoid. Before resecting this mass, due to bowel tethering, a colonoscopy was performed. The cecum was completely replaced by a carpeting villous adenoma. Two tubular adenomas and one tubulovillous adenoma, all with low-grade dysplasia, were noted.

During definitive surgery, the lesion arising from the sigmoid mesentery and suspected to be a desmoid, was shown to be a well-differentiated adenocarcinoma with endometrioid features. 28 tubulovillous adenomas and hyperplastic polyps, with no malignancy or dysplasia were identified in the subtotal colectomy. The clinical impression was that of attenuated polyposis and metastatic endometrial carcinoma.

## Results

APC gene testing was negative. This patient was homozygous for the common c.536A>G mutation in MYH.

## Conclusions

There remains controversy regarding the association of endometrial cancer and MAP. In 276 European MAP patients, the incidence of endometrial cancer was not significantly increased.[1] There are 3 reported cases of MAP patients diagnosed with endometrial cancer [2,3] , in addition to our patient. The patients in the literature were diagnosed at ages 46 (multiple adenomatous polyps detected at surgery), 61 (suspected FAP: subtotal colectomy at 41 years for polyps) and 53 years.

Although there is one case report of an individual with MAP having a desmoid[4], in the European cohort of 276 individuals with MAP above[1] , no desmoids were noted and in another group of 17 MAP patients, no desmoids were reported[5]. The individual with the desmoid [4] had an aggressive phenotype including 4 colon adenocarcinomas and over 100 colon polyps by age 30.

Given the growing literature regarding endometrial cancer in MAP and incomplete data regarding frequency of desmoid tumours in MAP, we would propose including MAP in your differential diagnosis when endometrial cancer, or an apparent desmoid are noted in association with multiple adenomatous polyps.
